# Low-Voltage and High-Reliability RF MEMS Switch with Combined Electrothermal and Electrostatic Actuation

**DOI:** 10.3390/mi12101237

**Published:** 2021-10-12

**Authors:** Yong Zhu, Jitendra Pal

**Affiliations:** 1 Queensland Micro and Nanotechnology Centre, Griffith University, Nathan, QLD 4111, Australia; 2 Wispry Inc., Irvine, CA 92618, USA; jpiitr84@gmail.com

**Keywords:** microelectromechanical systems (MEMS), radio frequency (RF), switch, electrothermal actuation, electrostatic actuation, insertion loss, isolation

## Abstract

In this paper, we report a novel laterally actuated Radio Frequency (RF) Microelectromechanical Systems (MEMS) switch, which is based on a combination of electrothermal actuation and electrostatic latching hold. The switch takes the advantages of both actuation mechanisms: large actuation force, low actuation voltage, and high reliability of the thermal actuation for initial movement; and low power consumption of the electrostatic actuation for holding the switch in position in ON state. The switch with an initial switch gap of 7 µm has an electrothermal actuation voltage of 7 V and an electrostatic holding voltage of 21 V. The switch achieves superior RF performances: the measured insertion loss is −0.73 dB at 6 GHz, whereas the isolation is −46 dB at 6 GHz. In addition, the switch shows high reliability and power handling capability: the switch can operate up to 10 million cycles without failure with 1 W power applied to its signal line.

## 1. Introduction

The increasing demand for high-performance, low-cost, and miniaturized wireless communication systems has driven the rapid development of novel devices for radio frequency (RF), microwave, and millimeter-wave circuits and systems. RF switches are essential devices to handle RF signal in these systems. Conventionally, RF switching has been done using semiconductor devices such as positive-intrinsic-negative (PIN) diodes and field-effect transistors (FETs) [1,2,3]. However, these semiconductor switches inherently suffer high losses at high frequencies in GHz range. Many researchers have attempted to investigate potential RF switching devices which are compact, linear, and power efficient. RF microelectromechanical systems (MEMS) are one of the potential candidates which have reformed the RF systems by realization of high-performance devices and components. For instance, RF MEMS switches have superior electrical performance compared to the traditional solid-state solutions, e.g., higher linearity, higher isolation, lower insertion loss, smaller size, and lower cost [4,5].

To achieve high isolation, large separation gap in OFF state is needed in RF transmission line. Currently, electrothermal and electrostatic actuators are widely used to generate large separation displacement in MEMS switches. Electrothermally actuated devices have simple fabrication processes, require lower actuation voltage, and are suitable for an application which demands higher contact force [6,7]. However, electrothermal actuation consumes high power which limits it from some applications where devices need to maintain ON position for a significant period of time [8]. By contrast, electrostatic actuated devices consume nearly zero low power as no DC current is needed during the actuation process. However, electrostatic based devices are limited by the trade-off between operating voltages and mechanical properties of the device [9]. For example, the operating voltage required is limited by the available voltage on the device in the whole RF system, which may be typically low (e.g., 5 V). However, high operating voltage, typically more than 50 V is required to reduce the parasitic load of the RF signal line, avoid a self-latching, and have a large mechanical separation between input and output ports.

In addition, RF MEMS switches mainly adopt metal-to-metal contact and capacitive coupling for the switching operations. The metal-to-metal contact requires good ohmic contact between two metal electrodes, whereas capacitive coupling switches have a thin dielectric film and an air gap between two metal electrodes. However, the above-mentioned contact topologies both suffer long-term reliability issues, thereby limiting their practical use in commercial applications [10,11]. For example, the issues include the dielectric charging due to large contact areas in capacitive coupling switch [12], and the stiction due to low restoring spring force in the metal contacting switches [13].

To solve the above issues, we propose a new design of MEMS switches, combining the unique characteristics of electrothermal and electrostatic actuators. This can avoid the high-power consumption issue in an electrothermal actuator as well as high-voltage issue in an electrostatic actuator [14,15,16,17]. Previously, a similar concept has been used in [18,19,20,21]: these designs utilize electrothermal actuators to bring the switch contacts near to signal lines and then hold the switches in ON state with electrostatic actuation. In another attempt, the switches are also integrated with electrostatic and electromagnetic actuation to achieve similar advantages [22,23]. However, they are all vertically actuated (out-of-plane) which require special fabrication process, thereby making the overall process complicated and the device less reliable. Compared to those vertically actuated MEMS switches, the laterally actuated MEMS switches have the advantages of in-plane design flexibility, high mechanical stability and reliability, and simple process. In addition, the actuators and the RF structures can be fabricated in a single lithographic step which dramatically reduces the fabrication cost and improves the production yield. Besides, it is easy to get a large separation, large contact area, and restoring force under a low voltage.

In this paper, we report a lateral actuated RF MEMS switch that employs a two-step switching operation: a V-shaped electrothermal actuator moves the switch contacts to touch the separated RF signal lines (close the switch); and then the switch remains in the ON state using electrostatic latching mechanism. The fabricated device avoids the constant power consumption requirement for electrothermal actuator and the high voltage requirement for electrostatic actuator when large gaps are required. Hence, power is consumed only during the electrothermal actuation for a short period and the switch consumes almost zero power by electrostatic latching in the holding period.

This paper is organized as follows. In Section 2, we demonstrate the design concept and operation of the proposed MEMS switch. This section also discusses simulation results using Intellisuite and the fabrication process for the proposed design. Section 3 then presents the measurement results and discussion. Finally, conclusion is presented in Section 4.

## 2. Materials and Methods

### 2.1. Design

**Figure 1:**
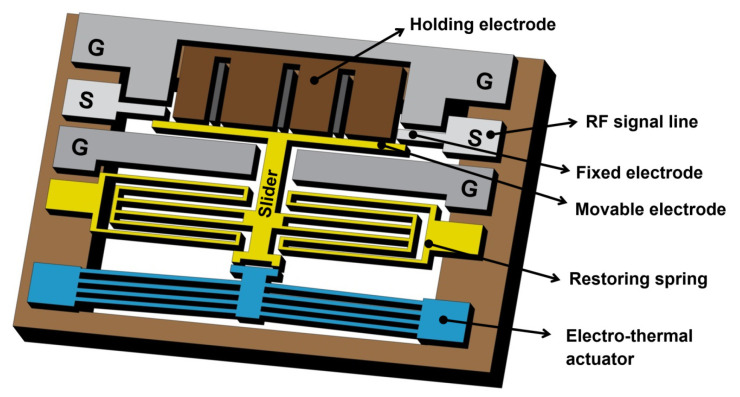
Schematic diagram of the proposed microelectromechanical systems (MEMS) switch design with combined electrothermal actuation and electrostatic hold.

The schematic diagram of the proposed design is shown in Figure 1. The switch consists of five main parts including Co-planar waveguide (CPW), fixed electrode, movable electrode (slider), V-shaped electrothermal actuator, and electrostatic holding electrodes. The movable slider (electrode) changes the switch states between ON and OFF by connecting and disconnecting contact tips in the separated RF signal line. A V-shaped electrothermal actuator is used to drive the movable electrode to close the switch, and then the electrostatic holding electrodes hold the movable electrode in contact position of ON state. The design has the combined advantages of low actuation voltage from electrothermal actuator and low power consumption in ON state due to electrostatic latching. In addition, the switch offers large initial gap of 7 µm between signal line and movable electrodes, which provides high isolation benefit that is difficult to achieve in electrostatic actuation. To increase reliability and power-handling capability, the switch is designed with high spring constant which restricts the switch from stiction issue and self-actuation issue. Moreover, the air gap between the slider and the actuator prevents the heat transferring from actuator to RF signal line.

**Figure 2:**
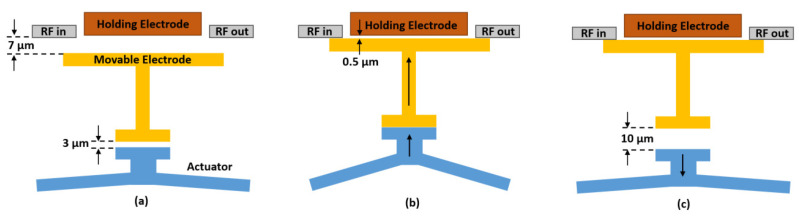
Switching mechanism and steps: (**a**) initial OFF state; (**b**) intermediate state with actuation voltage applied to electrothermal actuator to close the switch; (**c**) ON state latched by electrostatic force from the holding electrode.

The switching mechanism and steps of proposed design are shown in Figure 2. During switching states, the main acting forces are: (1) thermal expansion force, (2) spring force, and (3) electrostatic force. In the initial step (see Figure 2a), a large air gap of 7 µm exists between the RF signal line and the switching electrode (slider) to make the switch in OFF state with good isolation. Then electrical current follows through the V-shaped electrothermal actuator, thermal expansion force pushes the slider to close the air gap and contact with the transmission line, shifting the switch into ON state (see Figure 2b). Next, the voltage applied to the holding electrode generates electrostatic force between fixed electrode and movable electrode. The electrostatic force is sufficiently high to hold the switch in ON state, so the applied current in the thermal actuator can be removed to reduce the power consumption (see Figure 2c). The switch can finally return to its initial position (OFF state) by the spring’s restoring force when the applied voltage on holding electrode is removed. In the whole operation, the electrostatic force is only operated during the electrostatic latching when the gap between fixed electrode and moving electrode is very small (500 nm), therefore the switch requires low operating voltage. In addition, electro-thermal force is only needed for a short period during switching states to move the movable electrode to touch the fixed electrode, thus the ON state does not consume power. Therefore, the proposed design can be implemented in the applications where both low actuation voltage and low power consumption are required.

### 2.2. Modeling and Simulation

**Figure 3:**
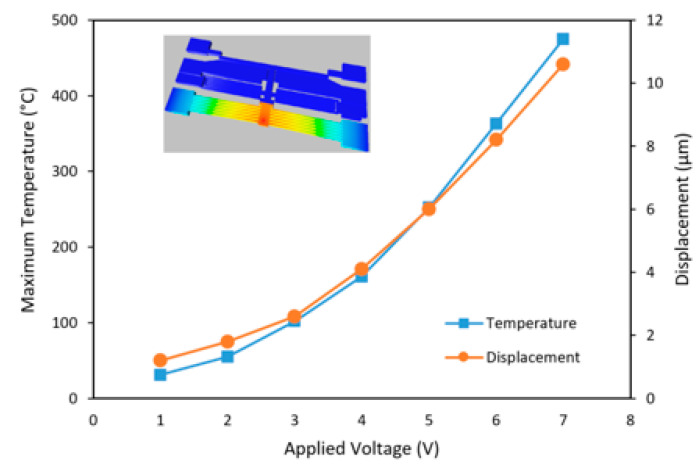
The simulated maximum temperature and displacement of the electrothermal actuator with various applied actuation voltages.

To analyse the performance and optimize the proposed design, finite element method (FEM) simulation is conducted using IntelliSuite’s Coupled Thermoelectromechanical Solver (IntelliSense Inc., Lynnfield, MA, USA). The material properties and optimized dimensions of the switch are summarized in Table 1. The initial state of the switch is OFF because the movable beam does not contact the signal line (with an initial air gap of 7 µm). When electric current flows through the V-shaped beams, the electrothermal actuator generates movement due to the thermal expansion of its material. As a result, the movable electrode moves upward and touches the broken signal lines (see Figure 2b). Figure 3 shows the simulated corresponding curve between maximum temperature and various applied actuation voltages from 1 to 7 V. The results show that the maximum temperature in the thermal actuator is 475 °C, which is well below the melting point of silicon. To provide thermal and electrical isolations from the silicon substrate, the device is trenched completely underneath the movable structures. Figure 3 also plots the simulated corresponding curve of displacement with applied voltage. The switch provides large displacement with much lower voltage compared with electrostatic actuation counterparts [9].

**Figure 4:**
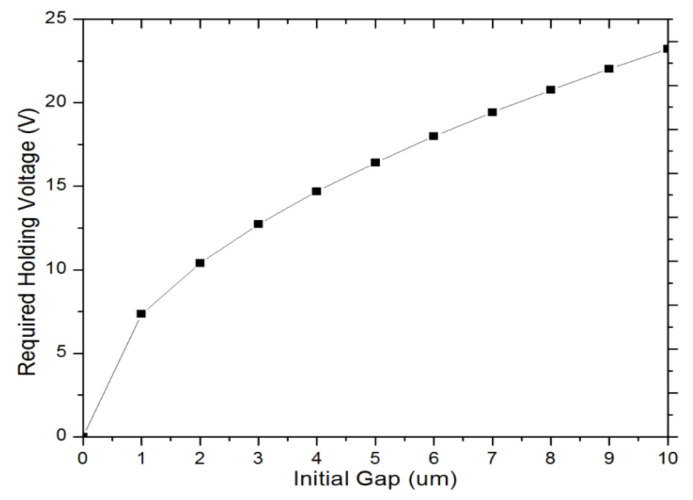
Required electrostatic holding voltages versus various initial gaps between switching electrodes.

When the movable beam touches the broken signal lines, a holding voltage *V* is applied between fixed electrode and movable beam to hold the movable electrode in position (see Figure 2c). Then the electric current in the V-shaped actuator is removed to reduce the power consumption. The electrostatic holding force $F_{hold}$ generated by the holding electrode can be estimated by

(1) $$F_{hold}=\frac{\varepsilon AV^{2}}{2g^{2}}$$

where ε is the dielectric constant of air; *A* and *g* are the overlap area and final gap between the holding electrode and movable electrode, respectively. When the electrostatic holding force *F_hold_* and the restoring force of the suspension spring are equal, the holding voltage can be defined as

(2) $$V_{hold}=g\sqrt{\frac{2kd}{\varepsilon A}}$$

where *d* is the initial gap between switching electrodes and *k* is the spring constant of the suspension beam. As Equation (2) implicated, a low holding voltage can be obtained by manipulating the parameters: *k*, *d*, *g,* and *A*. In our design, the spring constant *k* is 19.81 N/m (simulated using Intellisuite), initial gap *d* is 7 µm, overlap area *A* is 830 × 25 µm^2^, and final gap *g* is 0.5 µm. According to Equation (2), the required holding voltage is 19.4 V. The holding voltage can be reduced further by increasing the overlap area or decreasing the spring constant. However, larger overlap area will increase the overall device size, while smaller spring constant will make the device less robust to mechanical shock. As shown in Equation (2), initial gap *g* is outside of the square root operation, which means it is more significant in changing *V_hold_* values compared to other parameters. Figure 4 plots the corresponding curve between required holding voltage *V_hold_* and initial gap *g*. When the initial gap varies from 1 µm to 10 µm, the required holding voltage changes from 7.34 V to 23.22 V, which is fairly low in comparison with typical actuation voltage (~100 V) in electrostatic actuation.

**Figure 5:**
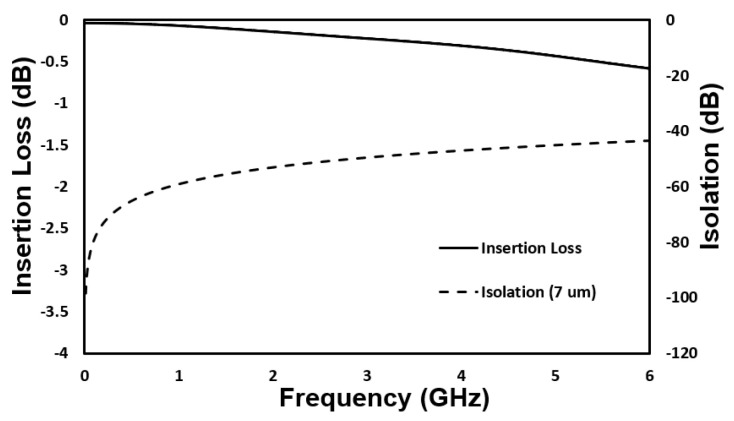
Simulated radio frequency (RF) performances of proposed switch in ON and OFF states.

To investigate and optimize the RF performance of the proposed switch, the finite integration technology has been utilized in CST Microwave Studio. The coplanar waveguide (CPW) transmission line is set as gap/width/gap (G/W/G) of 55/100/55 µm for a 50 Ω impedance. The simulated results show very low insertion loss of −0.58 dB in ON state and very high isolation of −43.47 dB in OFF state at 6 GHz, as shown in Figure 5.

### 2.3. Fabrication

**Figure 6:**
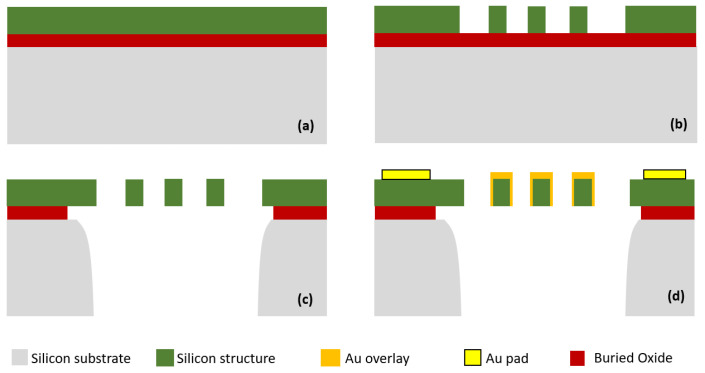
Fabrication process flow including four key steps: (**a**) starting SOI wafer, (**b**) Si structure patterning by deep reactive ion etched (DRIE), (**c**) Structure releasing by backside DRIE dry etching and wet etching, and (**d**) Metal layers deposition for connection and overcoating.

**Figure 7:**
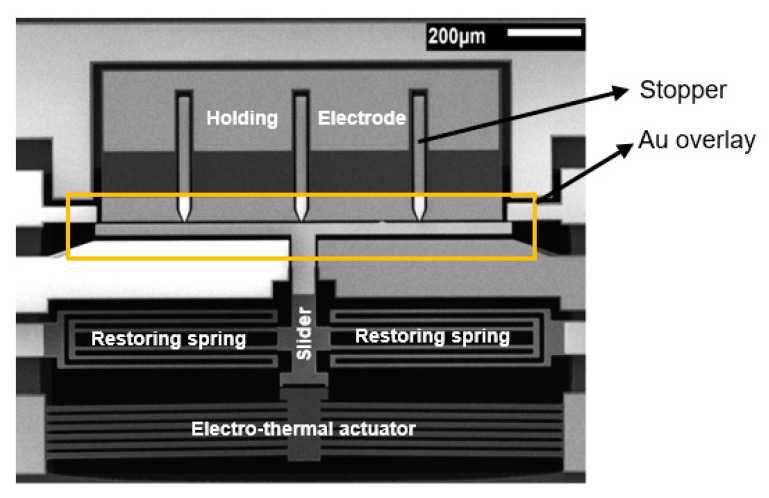
Scanning Electron Microscopy (SEM) photo of the fabricated switch. Au overlay is added to reduce the contact resistance and RF signal loss. Three stoppers ensure no contact and short circuit between movable electrode and holding electrode.

The proposed design was fabricated using a commercially available SOIMUMPS process [24]. The process starts with a silicon-on-insulator (SOI) wafer consisting of a 25 µm silicon layer, a 2 µm oxide layer, and a 400 µm substrate layer as shown in Figure 6a. To define the mechanical structure, the top silicon layer is lithographically patterned, and deep reactive ion etched (DIRE) (see Figure 6b). Next, trenches are made by selectively backside etching the wafer through the substrate layer (see Figure 6c). The advantages with these trenches include releasing the movable structures in the top silicon layer, and reducing the substrate parasitics, thereby improving the RF performances. In addition, the SOIMUMPs offers two metal layers: (1) the pad metal layer which consists of a 20 nm chrome and 500 nm of gold for the electrical connection; and (2) the blanket metal layer consisting of 600 nm Au and 50 nm Cr. The blanket metal layer partially overcoated on the silicon structure, which can reduce the RF signal loss in the signal line as well as reduce the switch contact resistance. The pad metal layer is patterned through a lift-off process whereas the blanket metal layer is deposited using a shadow mask technique to reduce the contact resistance (see Figure 6d). To further reduce the contact resistance, a 1 µm Cu is over-coated on the top and sidewall of the switching area by sputtering process [25,26,27]. Figure 6 and Figure 7 illustrate the fabrication process flow with the key steps and SEM image of the fabricated switch, respectively.

## 3. Measurements and Discussion

### 3.1. Direct Current (DC) and Transient Measurements

**Figure 8:**
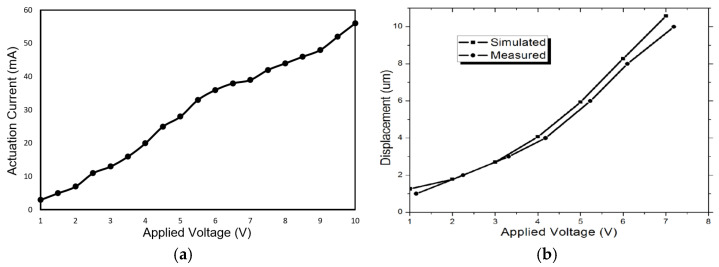
(**a**) Measured I–V characteristics of the fabricated V-shaped electrothermal actuator. (**b**) Comparison between simulation and measured displacements vs. applied actuation voltages.

To characterize the actuation behaviour, the direct current (DC) electromechanical response measurement was implemented. First, we measured the current as a function of applied voltage for the actuator and results are shown in Figure 8a. Approximated linear response has been obtained with an actuator resistance of 178.6 Ω and a power consumption of 273 mW at the actuation voltage of 7 V. Figure 8b plots the curve of displacement with varying applied actuation voltage. As expected, the displacement increases with the increase in voltage and the measured results match the simulation results well. To reduce the thermal conduction to the substrate, air trench was made underneath the actuator (see Figure 6). As a result, the improved electrothermal actuation efficiency helped the actuator achieve large displacement of 10 µm under an actuation voltage of 7 V, as shown in Figure 8b. It should be noted that a total of 10 µm actuation displacement is needed to close the switch, which includes 3 µm gap of actuator/movable electrode and 7 µm gap of holding electrode/movable electrode, as shown in Figure 2a–c.

**Figure 9:**
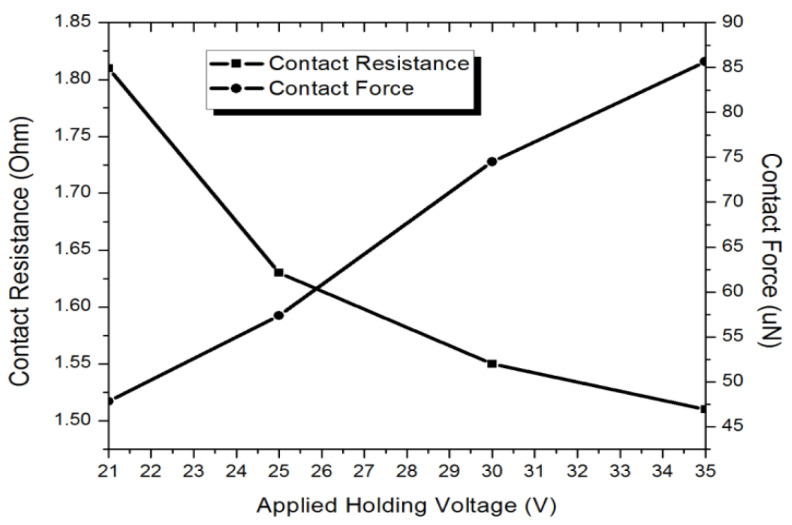
Changes in measured contact resistance and simulated contact force with various applied holding voltages.

Figure 9 illustrates the relationship between contact resistance and applied holding voltage after the switch is closed. The testing result showed that the fabricated switch requires a minimum holding voltage of 21 V for the switch with initial gap 7 µm, which matches well with the calculated results of 19.4 V using Equation (2). The small discrepancy is believed to be due to the fabrication process uncertainties. The holding voltage is applied through a DC voltage source and the switch contact resistance is continuously observed with a digital multimeter. When an applied holding voltage of 21 V is applied between movable and fixed electrodes, the digital multimeter shows a contact resistance of 1.81 Ω. When the applied holding voltage increases from 21 V to 35 V, the contact resistance reduces to 1.51 Ω. This is because the contact force increases with the increase of the applied holding voltage as shown as the simulated electrostatic contact force results in Figure 9. The contact pressure was firstly simulated at various applied voltages using Intellisuite software. Then the contact force was calculated by multiplying contact pressure with the contact area.

**Figure 10:**
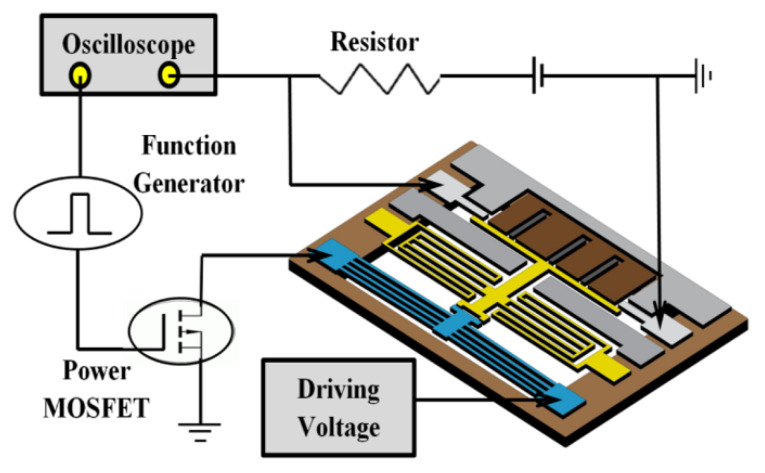
The setup schematic for measuring switching times.

**Figure 11:**
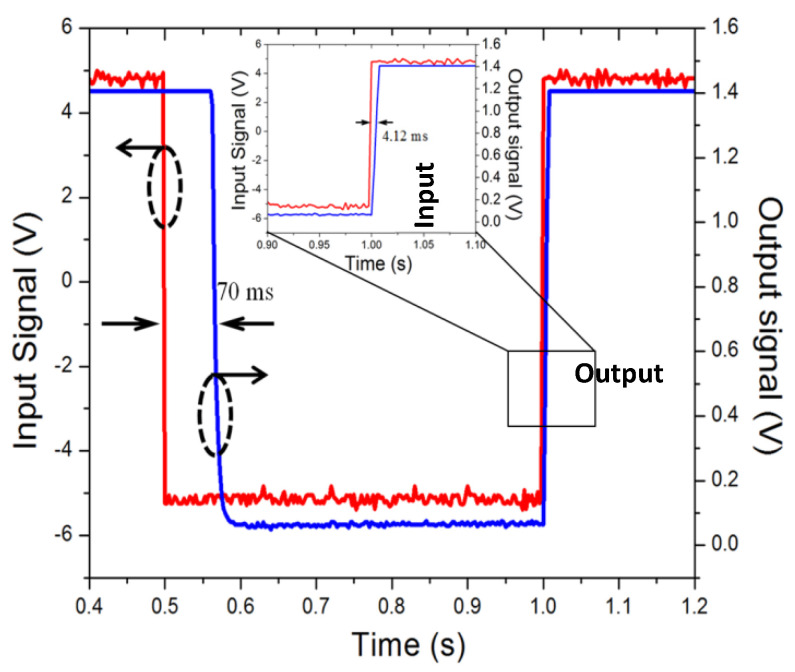
The measured transient switching waveforms of the fabricated switch.

Figure 10 illustrates the measurement set-up schematic for measuring the switching times. As the switching drive signal, a 1 Hz square wave voltage signal (5 Vpp and 50% duty cycle) is supplied by a function generator. A power MOSFET is used to separate the input control signal from the switch driving voltage signal. The driving voltage is supplied with a constant power supply of 1.5 V. One port of switch is grounded and the other port is connected to an oscilloscope to monitor the transient switching times. Figure 11 plots the measured switching times of the fabricated switch. The switch has shown a response time of 70 ms for the transition from open state to close state, and a releasing time of 4.12 ms for the transition from close state to open state. The measured response time is much longer than the releasing time. This is due to the fact that the movable electrode needs to travel a total distance of 10 µm before contacting the broken signal line, whereas the disconnection of the switch only needs to separate the movable beam from signal lines by removing the applied drive voltage. It should be noted that the switching speed of the electrothermal actuator is slower compared to that of its electrostatic counterparts. The reason is that electrothermal actuator generates its movement through Joule heating effect, which is inherently slower than electrostatic effect.

### 3.2. RF Characteristics

**Figure 12:**
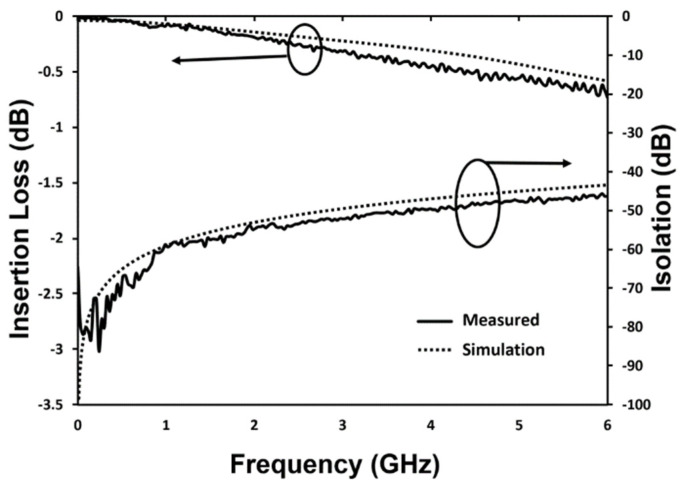
Comparison between the measured and simulated RF performances (Insertion Loss and Isolation) of the fabricated switch.

The RF performance of the fabricated MEMS switch is characterized using a RF Vector Network Analyzer (FieldFox™ N9923A, Keysight, Santa Rosa, CA, USA) with ground-signal-ground (GSG) coplanar probes. The system is calibrated using the standard short-open-load-through (SOLT) on-wafer technique. The S-parameters of the input and output ports are extracted using the Vector Network Analyzer. In the measurement setup, input, and output ports of the CPW are connected with the network analyser, while the actuation electrodes are connected with the DC voltage source. When a voltage is applied to the fabricated switch for actuation, the S parameter is measured through input and output ports as shown in Figure 12. In OFF state, the performance of the switch is represented by isolation. The measured isolation is better than −46 dB at 6 GHz. The high isolation for the fabricated switch is achieved due to large initial gap of 7 µm between signal line and the movable electrode. The measured insertion loss in ON state has a maximum value of −0.73 dB at 6 GHz. The insertion loss includes the losses due to the contact resistive loss, long CPW lines loss and radiation loss due to discontinuity in CPW structure [25]. The RF characteristics of fabricated switch are well matched with simulation results. The small discrepancy between the simulation and measurement results is believed to be caused by fabrication process uncertainties.

### 3.3. Reliabilty Test

In MEMS switches, the limitations on lifetime come mainly from hot switching rather than cold switching. The electrostatically actuated switch generally suffers from the mechanism of self-switching and RF latching. In contrast, the electrothermally actuated switches suffer mainly from micro-welding between the contacts. Our electrothermal switch is expected to be independent from these issues because of their high mechanical restoring force. As a result, the main limitations come from the RF current and contact degradation.

**Figure 13:**
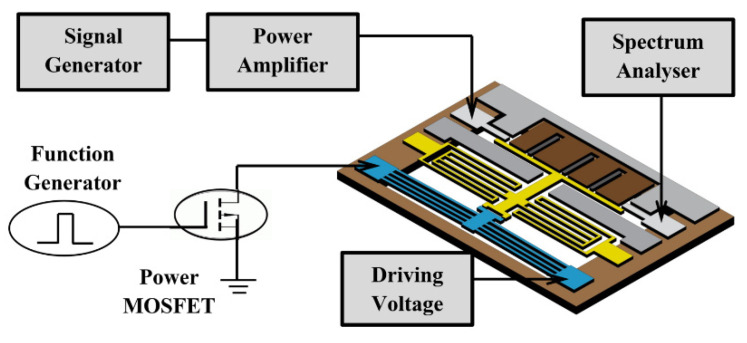
The test setup schematic for measuring RF power handling capability of the fabricated switch.

In this paper, the hot switching test is done under high RF power. Figure 13 illustrates the measurement setup to measure the RF power handling capability of fabricated switch. To estimate the self-actuation power, an input power signal of 1 W is applied to the fabricated device which is generated by signal generator and power amplifier. The switch does not show any self-actuation phenomenon when a power of 1 W is applied, therefore electrostatic force generated by RF voltage between signal lines and movable beam is negligible. To observe micro-welding phenomenon through contact, RF signals at 500 MHz with powers of 1 W is applied to contacts. The result shows that the switch can handle up to 10 million cycles when a power level of 1W is applied to the switch. The test was stopped after 10 million cycles not due to switch failure.

**Figure 14:**
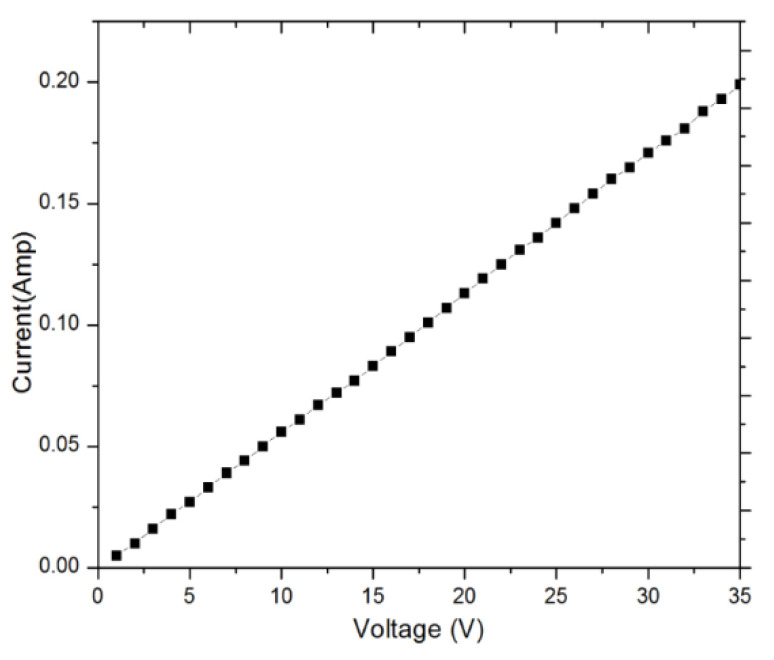
Voltage versus current in hot switching test over a 100 Ω resistor.

The contact reliability of the fabricated switch in terms of switching cycles under high voltage is also investigated [28]. The test is conducted in air ambient and hot switching condition with high applied voltage from a dc power supply across a resistive load. The applied voltage is continuously increased in steps of 1V until the contact survives. The fabricated switch can handle hot switching up to 35 V and 0.2 A across a 100 Ω resistor as shown in Figure 14. When voltage is further increased, the switch failed in open state and contact resistance jumps to several mega-ohms. The mechanisms of failure include deterioration of contact surface and deformation of silicon beams. The other possibility of switch failure could be airborne contamination as the measurement was conducted under atmospheric conditions without hermetic packaging. The reliability of the switch can be improved by using hard materials such as Platinum (Pt) and Ruthenium (Ru) instead of commonly used Au-to-Au contact [29,30,31]. Another way to increase the power handling capability is to reduce the current through each contact by implementing multiple contacts in the switch [32,33,34]. In addition, hermetic packaging can also improve the switch reliability for its protection from humidity and contamination.

## 4. Conclusions

In this paper, a novel low-voltage and high-reliability switch actuated with the combination of electrostatic and electrothermal actuation is designed, fabricated, and characterized. By using the combined actuation mechanisms, the switch can achieve a low actuation voltage of 7 V and a low holding voltage of 21 V. The proposed switch exhibits excellent RF performances in terms of insertion loss and isolation. The measured insertion loss and isolation is −0.7 dB and −46 dB at 6 GHz, respectively. The switch did not show any self-actuation phenomenon with the input power of 1 W. The hot switching results indicate that the switch can handle high power up to 1 W for 10 million cycles without failure. Although the switching time of the proposed fabricated switch is longer compared to electrostatic actuation, the fabricated switch can be used in the application where longer switching time can be affordable such as multiband receiver band selection networks, redundancy networks, etc. It is noted that this paper is focused on a novel concept for low voltage and low power consumption, and robustness tests will be investigated in future studies.

## Figures and Tables

**Table 1:** Material properties of the bulk silicon used in the switch and the optimized device dimensions.

Properties and Dimensions	Name	Value	Unit
Mechanical Properties	Young’s modulus	170	GPa
	Poisson’s ratio	0.28	-
	Density	2.3	g/cm^3^
Thermal Properties	Thermal conductivity	1.46	W/cm·K
	Thermal expansion coefficient	2.7 × 10^−6^	K^−1^
Electrical Properties	Electrical resistivity	0.013	ohm·cm
Geometry Dimensions of the Designed Switch Structures	Length of V-shaped actuator beams (each side)	400	µm
	Width of V-shaped actuator beams	10	µm
	Angle of V-shaped actuator beams	0.6	°
	Number of V-shaped actuator beams (each side)	6	-
	Length of Suspension spring (each side)	470	µm
	Width of Suspension spring (each side)	5	µm
	Device thickness	25	µm
	Initial gap between switching electrodes, *d*	7	µm
	Holding electrode gap, *g*	0.5	µm
	Holding electrode overlap area, *A*	830 × 25	µm^2^
	Switch contact area (each side)	6.5 × 25	µm^2^
