# Erratum: Zhu, Y.; Pal, J. Low-Voltage and High-Reliability RF MEMS Switch with Combined Electrothermal and Electrostatic Actuation. *Micromachines* 2021, *12*, 1237

**DOI:** 10.3390/mi12111389

**Published:** 2021-11-12

**Authors:** Yong Zhu, Jitendra Pal

**Affiliations:** 1 Queensland Micro and Nanotechnology Centre, Griffith University, Nathan, QLD 4111, Australia; 2 Wispry Inc., Irvine, CA 92618, USA; jpiitr84@gmail.com

The authors would like to update the Figure 3 and Figure 7 to the published paper [1] as follows:

The changes do not affect the scientific results. We apologize for any inconvenience caused to the readers by these errors. The manuscript will be updated with a reference to this Erratum.

## Figures and Tables

**Figure 3:**
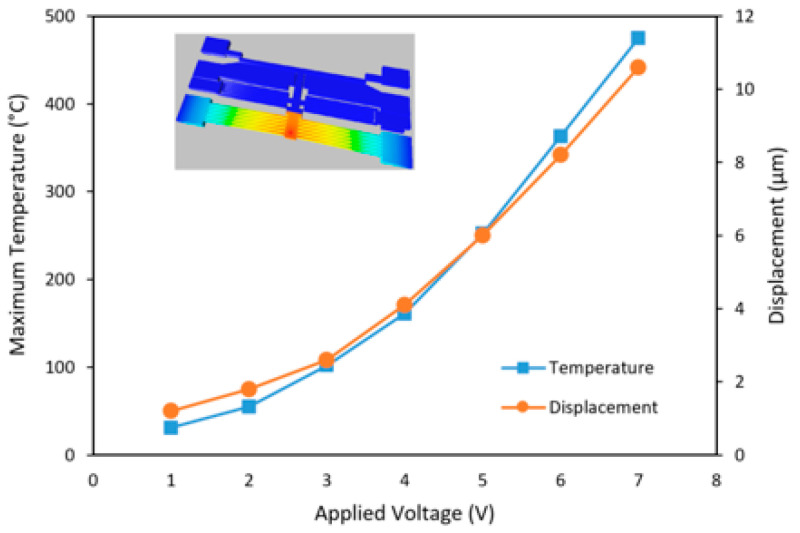
The simulated maximum temperature and displacement of the electrothermal actuator with various applied actuation voltages.

**Figure 7:**
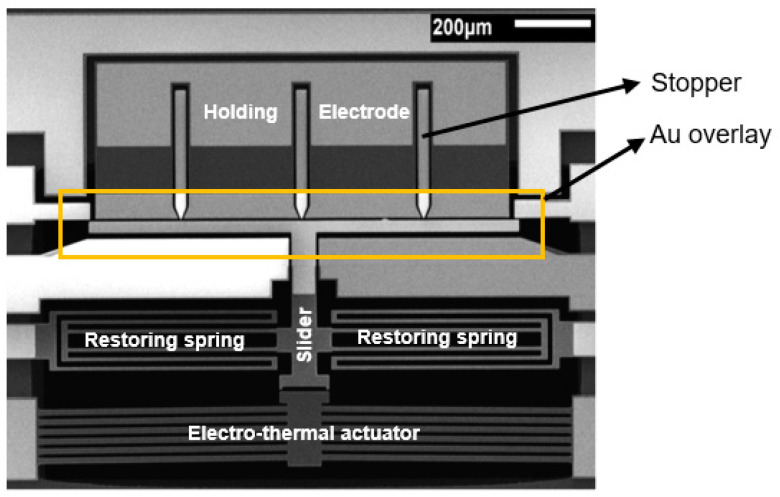
Scanning Electron Microscopy (SEM) photo of the fabricated switch. Au overlay is added to reduce the contact resistance and RF signal loss. Three stoppers ensure no contact and short circuit between movable electrode and holding electrode.
